# Vertical traction device prevents abdominal wall retraction and facilitates early primary fascial closure of septic and non-septic open abdomen

**DOI:** 10.1007/s00423-021-02424-1

**Published:** 2022-02-11

**Authors:** Stephen Fung, Hany Ashmawy, Christian Krieglstein, Thomas Halama, Dustin Schilawa, Oliver Fuckert, Anita Hees, Feride Kröpil, Alexander Rehders, Nadja C. Lehwald-Tywuschik, Wolfram Trudo Knoefel

**Affiliations:** 1 Department of Surgery (A), Heinrich Heine University and University Hospital Duesseldorf, Moorenstrasse 5, 40225 Duesseldorf, Germany; 2 Department of Surgery, St. Elisabeth Krankenhaus Köln, Werthmannstr. 1, 50935 Cologne, Germany; 3 Department of Surgery, St. Vinzenz-Hospital, Merheimer Str. 221-223, 50733 Cologne, Germany; 4 Department of Surgery, St. Rochus Krankenhaus, Glückaufstraße 10, 44575 Castrop-Rauxel, Germany; 5 Department of Surgery, Lukas Krankenhaus, Hindenburgstraße 56, 32257 Bünde, Germany; 6 Department of Surgery, St.-Marien Krankenhaus, Kampenstraße 51, 57072 Siegen, Germany

**Keywords:** Vertical traction, Open abdomen, Retraction prevention, Fasciotens

## Abstract

**Purpose:**

One of the major challenges in the management of patients with septic and non-septic open abdomen (OA) is to control abdominal wall retraction. The aim of this study was to evaluate the impact of a novel vertical traction device (VTD) on primary fascial closure (PFC) and prevention of fascial retraction.

**Methods:**

Twenty patients treated with OA were included in this retrospective multicenter study. All patients were initially stabilized with laparostomy and the abdomen temporarily sealed either with a Bogotá bag or a negative pressure wound therapy system (NPWT).

**Results:**

The mean duration of OA and fascia-to-fascia distance (FTF) prior to the VTD application were 3 days and 15 cm, respectively. At relook laparotomy 48 h after VTD implementation, the mean FTF distance significantly decreased to 10 cm (*p* = 0.0081). In all cases, PFC was achieved after a mean period of 7 days. Twelve patients received the VTD in combination with a NPWT, whereas in eight patients, the device was combined with an alternative temporary abdominal closure system (TAC). Although not statistically significant, the FTF distance remarkably decreased in both groups at relook laparotomy 48 h following the device implementation. The mean periods of PFC for patients with septic and non-septic OA were comparable (7.5 vs. 7 days). During follow-up, two patients developed an incisional hernia.

**Conclusion:**

Vertical traction device prevents fascial retraction and facilitates early PFC in OA. In combination with NPWT, rapid fascial closure of large abdominal defects can be achieved.

## Introduction

Septic peritonitis (SP), abdominal compartment syndrome (ACS), and damage control surgery (DCS) often lead to open abdomen (OA) treatment [1, 2]. In most cases, laparostomy is a life-saving surgical procedure. Patients with OA are critically ill and are often susceptible to multiple organ dysfunctions [3]. Particularly when prolonged, OA has been reported to be associated with increased risk of bowel adhesions, entero-atmospheric fistulas, intra-abdominal abscesses, and formation of complex abdominal wall hernias due to loss of domain [4–8]. According to the World Society of Emergency Surgery (WSES) guidelines, early fascial closure should be the strategy for the management of OA once any requirements for ongoing resuscitation have ceased and the source control has been definitively reached [1]. Thus, early primary fascial closure (PFC) should be a main treatment goal of OA to mitigate morbidity and mortality [9].

However, early PFC of OA still remains a challenge. In the last decade, several techniques for temporary abdominal closure systems (TAC) of OA have been implemented including Bogotá bag, Wittmann Patch®, skin closure only, dynamic fascial traction devices, and negative pressure wound therapy (NPWT) [10–16]. PFC rates of various TAC techniques are heterogeneous and have been reported with 69–92% [4, 13, 17, 18]. Nowadays, the preferred standard technique for TAC of OA is the NPWT [1]. NPWT is widely reported to be associated with high fascial closure and low complication rates of non-septic OA (e.g., trauma patients) [2, 19–21]. However, several studies demonstrated that patients with septic OA (e.g., with peritonitis) have lower PFC rates compared with trauma patients [19, 20, 22]. Interestingly, combined with a dynamic closure procedure, NPWT has been observed to achieve higher closure rates compared with NPWT alone [15, 18, 23–25].

In the literature, the abdominal re-approximation anchor (ABRA) and the vacuum and mesh mediated fascial traction (VACM) have been described as effective dynamic closure procedures [13, 14, 18, 25, 26]. Both techniques exert a dynamic horizontal traction on the fascia, which can be re-approximated at bedside (ABRA) or in repeated abdominal explorations (ABRA/VACM).

The vertical traction device (Fasciotens® Abdomen) is a novel device, which exerts a dynamic vertical traction on the fascia. To date, this device has only been reported in preclinical trials to prevent fascial retraction during OA and to reduce the necessary traction force for fascial closure in a porcine model [27]. In a recent case report, the application of the vertical traction device (VTD) was reported for the first time in a single patient to enhance early PFC without mesh implementation or complex abdominal wall reconstruction [28]. However, this study represents the first clinical series with this novel device in patients with open abdomen.

The primary aim of this multicenter study was to evaluate the primary fascial closure (PFC) rates following the vertical traction device (VTD) implementation and to determine possible complications related to its clinical application. Secondly, we performed a subgroup analysis to investigate the clinical impact of vertical traction on fascial retraction and to evaluate the rates of primary fascial closure of patients with septic and non-septic open abdomen. Furthermore, the outcomes of vertical traction were investigated when combined with a negative pressure wound therapy or an alternative temporary abdominal closure system. Additionally, the underlying complications related with the device implementation were analyzed.

## Material and methods

### Patients

This retrospective multicenter study consisted of 20 patients treated between January 2019 and May 2020 with open abdomen (OA) at six different hospitals in Germany. The local ethic committee approved this study (study no: 2021–1319). In each center, the same surgeon performed the device application, supervised the device during clinical use, and performed the postoperative follow-up at the outpatient clinic. Causes of septic OA were gastrointestinal perforation (*n* = 10) and necrotizing, infected pancreatitis (*n* = 2). Non-septic OA resulted of ACS after aortic rupture and repair (*n* = 4), intestinal ischemia (*n* = 2), and mechanical ileus (*n* = 2). In all cases, the VTD was applied after hemodynamic control of the patient’s clinical condition. In most cases, a moderate catecholamine dose (norepinephrine) was applied to achieve hemodynamic stability. Eighteen patients were treated at the intensive care unit (ICU) and were under mechanical ventilation until the device was dismounted prior to primary fascial closure. Two patients were treated at the intermediate care unit (IMC) and were under a patient-controlled analgesia (PCA) prior to device application.

In twelve cases, the vertical traction device (VTD) was combined with a negative pressure wound therapy system (VTD-NPWT group) and in eight cases with an alternative temporary abdominal closure system (e.g., Bogotá bag) (VTD-TAC group). Patient demographic data were retrieved from medical records. Björck classification [29], fascia-to-fascia distance (FTF), APACHE II score, duration of OA, cause of OA, number of relook procedures until fascial closure, complications related to device application, and occurrence of incisional hernia during outpatient clinic follow-up were collected from documented data of the supervising surgeons.

### Device description

**Fig. 1:**
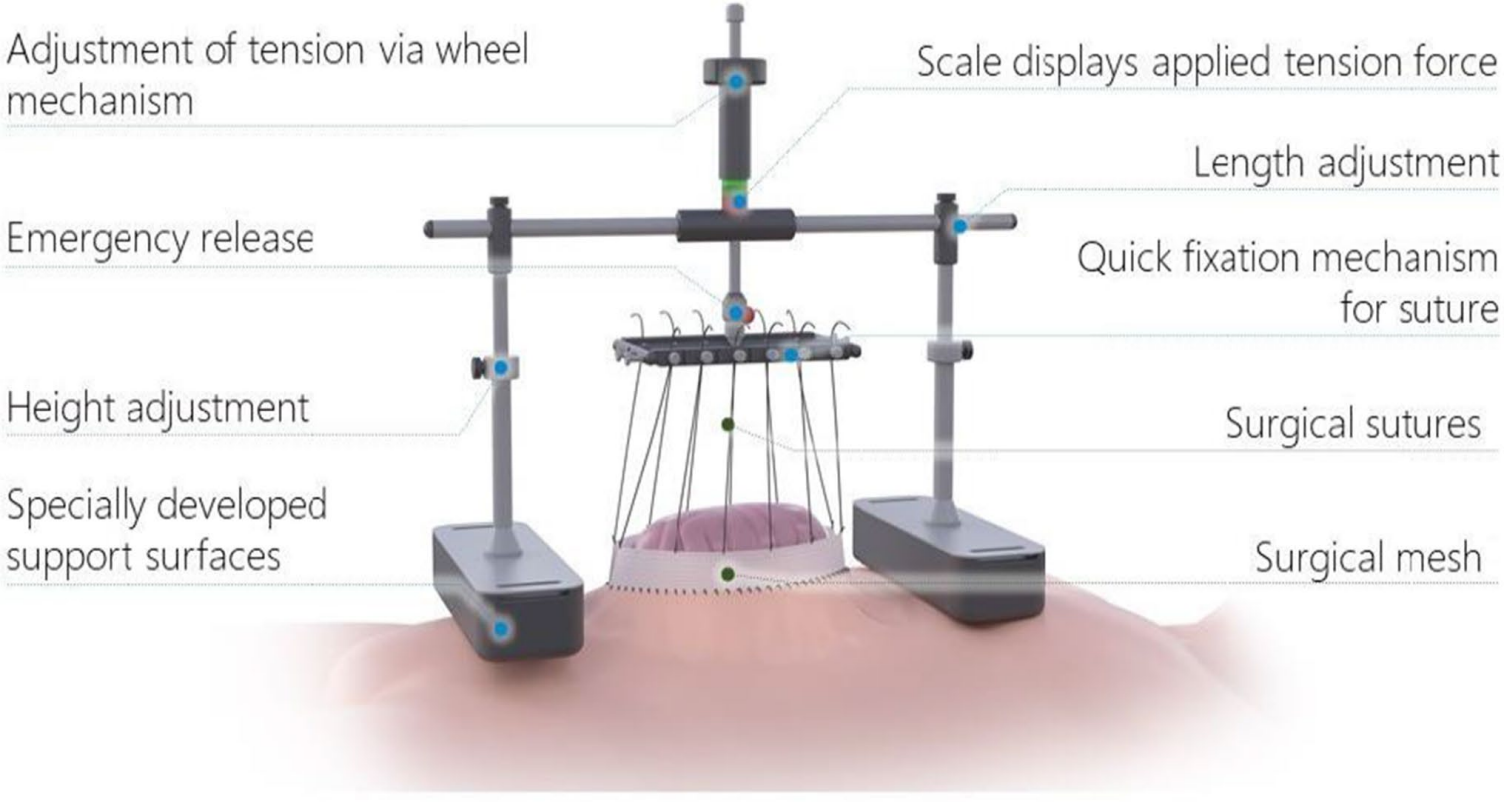
Set-up of the vertical traction device. Source of picture: Fasciotens® GmbH, Essen, Germany

**Fig. 2:**
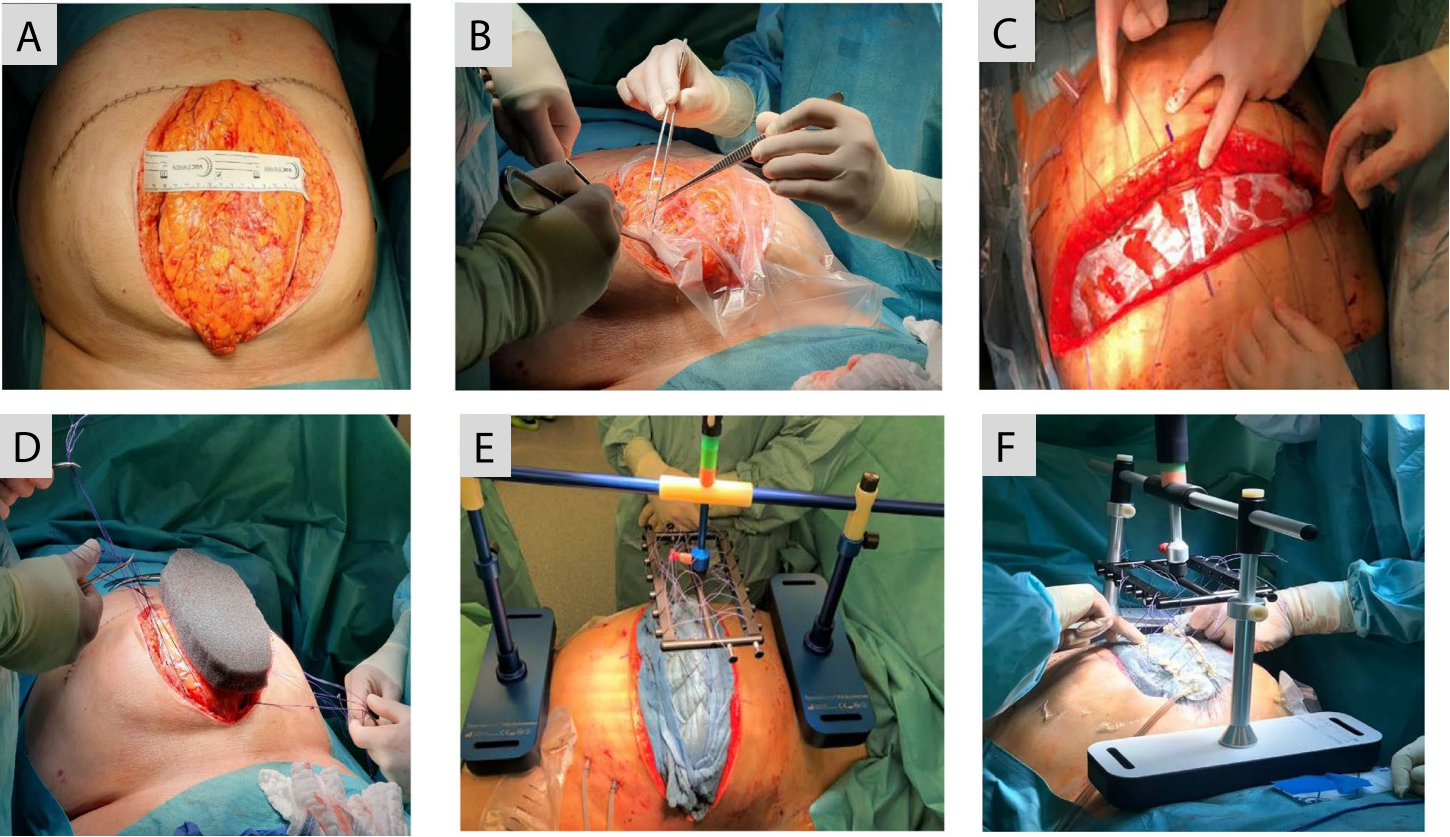
Device application on patient with open abdomen. **A** Measurement of fascia-to-fascia (FTF) distance at full patient relaxation. **B** Following lavage, the OA is sealed with a Bogotá bag. In this case, the patient was initially stabilized with a midline laparotomy. **C** Patient stabilized with transverse laparotomy. Abdomen sealed with a Bogotá bag and FTF distance measured at full patient relaxation. Six surgical sutures sewed on both edges of the vicryl meshes. **D** A doubled vicryl mesh is sewed on each fascial margin. Six surgical sutures are sewed on both edges of the vicryl meshes. V.A.C.® GRANUFOAM™ Dressing is placed on the Bogotá bag. **E** Vertical traction device with Bogotá bag and abdominal dressings (TAC). **F** Vertical traction device with negative pressure wound therapy applied

The vertical traction device (Fasciotens® Abdomen; Essen, Germany) description was derived from the study of Eickhoff et al. [27]. The main principle of the device is the application of dynamic vertical traction along both fascial margins over a clamping system (Figs. 1 and 2 E and F). The device consists of a beam with two buttresses positioned on the thorax and anterior pelvic ring. After midline or transverse laparotomy, a doubled vicryl mesh is sewed to each fascial margin using commercial sutures. Six sutures on the mesh of each fascial margin carried through eyelets are fastened on a common suspension. The eyelet suspension is attached to a longitudinal beam with a height-adjustable connection. Using this dynamic connection, the fascial traction can be increased or decreased as needed. The applied traction force is adjustable along a range of 0–100 N (Newton) as presented on the varying colored fields of the longitudinal beam. In our cases, we adjusted the fascial traction to the dark green field of the longitudinal beam. This field corresponds to a traction force of 60–80 N according to the manufacturer’s specifications. Once suspended and tensed on the adjustable longitudinal beam, the fascial margins are pulled vertically relative to the thorax and pelvis. This vertical traction withstands the natural muscle traction, counteracts resulting fascial retraction, and enhances anterior extensive tissue development. Simultaneously, the open abdomen allows pressure release. The treatment periods were approximately 5 h, followed by 1 h of treatment break. Over a period of 24 h, the dynamic traction force was applied for about 20 h. For the alert patient at the IMC unit, treatment breaks were undertaken according to the patient’s needs (< 20 h/24 h). As recommended by the manufacturers, this device was only used in patients under mechanical ventilation treated at the intensive care unit (ICU) and in patients at the intermediate care unit (IMC) with appropriate patient-controlled analgesia (PCA).

### Surgical technique

Prior to device application, the patient was intraoperatively fully relaxed with a muscle relaxant agent (e.g., Esmeron®), before the fascia-to-fascia (FTF) distance was measured (Fig. 2A). After abdominal lavage and surgical debridement of the fascia to create clean mobile fascial margins, a doubled vicryl mesh (maximum width 2–3 cm) was sewed on both fascial margins. Hereafter, twelve commercial surgical threads, with six sutured on each side to defined areas of both meshes (Fig. 2 C and D), were directly vertically or cross-tensed on the vertical traction device (VTD) clamping system. The abdominal cavity was sealed either with a Bogotá bag and abdominal dressings (Fig. 2 B and E) or a negative pressure wound therapy (NPWT) system (Fig. 2 D and F). During repeated abdominal exploration 48 h after VTD implementation and during further re-exploration, the FTF distance was determined at full patient relaxation. To preserve fascial integrity, the doubled meshes sewed at both fascial edges were left in place as long as possible and only replaced when defects occurred, which impaired traction force. According to Eickhoff et al. [27], a significant reduction of the initial FTF distance was observed between 24 and 48 h after application of the device in a porcine model. For comparability, we used the values of FTF distance at relook operation 48 h after application of the VTD. During observation in the ICU and IMC unit, the traction force was controlled every 2–4 h and, if necessary, re-adjusted at the bedside to ensure steady dynamic traction of the fascia.

### Statistical analysis

The statistical analysis was performed with SPSS 25.0 (Statistical Package for Social Sciences; SPSS Inc., Chicago, IL, USA). All data are presented as mean value, range, and percentages. Mean values of continuous variables between groups were compared with Student’s *t*-test. Statistical significance was considered at *p* < 0.05.

## Results

Patient characteristics are summarized in Table 1. Twenty patients treated with OA were included in this study. Our patient collective consisted of sixteen males and four females with a mean age of 60 years (range 36–80 years) (Table 1). The mean APACHE II score was 20 (range 15–28). According to the OA classification of Björck [29], twelve patients were classified as grade 2B, and four patients each were graded 1A and 1B, respectively. Thus, as proposed by Björck [29], patients with grade 2B were classified as septic OA (*n* = 12), whereas those graded Björck 1A and 1B were considered non-septic OA (*n* = 8).

**Table 1:** Patient characteristics

	*n* (%)
Gender	
Male	16 (80%)
Female	4 (20%)
Mean age (range) years	60 (36–80)
Causes for OA treatment	
GI perforation	10 (50%)
NEC pancreatitis	2 (10%)
Intestinal ischemia	2 (10%)
Mechanical ileus	2 (10%)
ACS after aortic rupture and repair	4 (20%)
Fascia-to-fascia distance (cm)	
Before device application (range)	15.5 (8–23)
APACHE II score (range)	20 (15–28)
Björck classification of OA	
1A	4 (20%)
1B	4 (20%)
2B	12 (60%)

Unless otherwise specified, all data are presented as mean. *APACHE* Acute Physiology And Chronic Health Evaluation score, *NEC pancreatitis* necrotizing pancreatitis, *ACS* abdominal compartment syndrome, *GI* gastrointestinal. The classification of OA according to Björck was noted before the application of vertical traction device: grade 1A, clean OA without adherence between bowel and abdominal wall or fixity of the abdominal wall; grade 1B, contaminated OA without adherence/fixity; grade 2A, clean OA developing adherence/fixity; grade 2B, contaminated OA developing adherence/fixity; grade 3, OA complicated by fistula formation; and grade 4, frozen OA with adherent/fixed bowel, unable to close surgically, with or without fistula

For the total cohort, the mean period prior the vertical traction device (VTD) application was 3 days (range 0–14 days) (Table 2). The fascia-to-fascia (FTF) distance before VTD application was 15 cm (range 8–23 cm). However, at relook laparotomy 48 h after the device was implemented, the mean FTF distance significantly decreased to 10 cm (range 6–17 cm; *p* = 0.0081) (Table 4). In all cases (*n* = 20), definitive skin and PFC was achieved without the use of mesh or component separation with a mean period of 7 days (range 3–24 days). However, four patients (20%) developed a subcutaneous wound dehiscence 1 week after primary closure. In these cases, the fascia and muscle were not affected. After debridement of the subcutaneous tissue, negative pressure wound therapy (NPWT) was applied for 1 week until secondary skin closure was performed. Two patients developed a fascial dehiscence and were treated further with a NPWT. During follow-up, these patients developed an incisional hernia. During clinical use of the VTD, one patient (5%) developed stage one pressure sores according to Barczak et al. (intact but reddened skin for more than 1 h after pressure release) [30]. Further investigated parameters such as respiratory impairments and intra-abdominal hypertension (IAH) related to the device application were not observed. Moreover, no patient died during clinical course. One patient died related to a COVID-19 infection 4 months after discharge from hospital. The mean number of relook procedures until fascial closure in our patient cohort was four (range 1–7).

**Table 2:** Postoperative characteristics related to VTD application

	*n* (%)
Fascia-to-fascia distance (cm) 48 h at relook laparotomy after device application (range)	10 (6–17)
Duration of OA until device application; days (range)	3 (0–14)
Total duration of OA; days (range)	11 (5–28)
Duration of OA until fascial closure after device application; days (range)	7 (3–24)
Number of relook procedures until PFC; (range)	4 (1–7)
Successful early PFC	20 (100%)
Complications after skin and PFC	
Subcutaneous wound dehiscence	4 (20%)
Fascial dehiscence	2 (10%)
Vertical traction device related complications	
Pressure sores	1 (5%)
Intra-abdominal hypertension (IAH)	0 (0%)
Hemodynamic impairment	0 (0%)
Respiratory impairment	0 (0%)
In-clinic mortality	0 (0%)
Complications during follow-up	
Incisional hernia	2 (10%)
Mortality (due to COVID-19 infection)	1 (5%)
Follow-up time, months (range)	8.5 (6–19)

Data are presented as mean (range) and are based on the total patient cohort (*n* = 20). *OA* open abdomen, *PFC* primary fascial closure, *VDT* vertical traction device

In twelve patients, the vertical traction device (VTD) was applied in combination with a negative pressure wound therapy system (NPWT) (VTD-NPWT group), while in eight patients, the VTD was implemented with an alternative temporary abdominal closure system (TAC: Bogotá bag with abdominal dressings) (VTD-TAC group) (Table 3). The mean open abdomen (OA) duration and fascia-to-fascia (FTF) distance before VTD-NPWT application was 2 days (range 0–6 days) and 17.5 cm (range 13–23 cm), respectively, whereas mean duration of OA was 4.5 days (range 1–14 days) and the FTF distance 13 cm (range 8–15 cm) before VTD-TAC implementation (Tables 3). In both groups, a distinct reduction of FTF distance was evident at relook laparotomy 48 h after VTD application: 14 cm (range 7–14 cm) for the VTD-NPWT group and 9.5 cm (range 6–10 cm) for the VTD-TAC group (Table 4), respectively. The mean days to primary fascial closure (PFC) for patients in the VTD-NPWT group (7 days, range 3–24 days) were comparable to those with VTD-TAC (7.5 days; range 5–14 days). The mean duration of OA in both groups before and after implementation of the device was 10.5 days (range 5–30 days) for the VTD-NPWT group and 11.5 days (range 7–28 days) for the VTD-TAC group.

**Table 3:** Comparison of VTD-NPWT vs. VTD-TAC group

	VTD-NPWT (*n* = 12)	VTD-TAC (*n* = 8)	*p*-value
Fascia-to-fascia distance (cm)			
Before device application (range)	17.5 (13–23)	13 (8–15)	0.0532*
48 h at relook laparotomy after device application (range)	14 (7–17)	9.5 (6–10)	0.0408*
Björck classification of OA			
1A	4	0	
1B	4	0	
2B	4	8	
Duration of OA prior to device application; days (range)	2 (0–6)	4.5 (1–14)	*0.2895*
Duration of OA until fascial closure after device application; days (range)	7 (3–24)	7.5 (5–14)	*0.8977*
Total duration of OA; days (range)	10.5 (5–30)	11.5 (7–28)	*0.8556*

^*^A *p*-value < 0.05 displays statistical significance. *NPWT* negative pressure wound therapy, *OA* open abdomen, *TAC* temporary abdominal closure, *VTD* vertical traction device

**Table 4:** Comparison of fascia-to-fascia distance before VTD and at relook operation 48 h after VTD application

Characteristics	Distance (cm) before FA application	Distance (cm) 48 h at relook operation	*p*-value
All patients (*n* = 20)	15 (8–23)	10 (6–17)	0.0081*
VTD-NPWT (*n* = 12)	17.5 (13–23)	14 (7–17)	*0.0979*
VTD-TAC (*n* = 8)	13 (8–15)	9.5 (6–10)	*0.0675*
Septic OA (*n* = 12)	13.5 (8–23)	10 (6–17)	*0.1565*
Non-septic OA (*n* = 8)	17.5 (15–20)	14 (7–15)	*0.1105*

^*^A *p*-value < 0.05 displays statistical significance. *NPWT* negative pressure wound therapy, *OA* open abdomen, *TAC* temporary abdominal closure, *VTD* vertical traction device

**Table 5:** Comparison of septic vs. non-septic OA

	Septic OA (*n* = 12)	Non-septic OA (*n* = 8)	*p*-value
Age (range); years	60 (36–65)	66 (54–80)	*0.4184*
Fascia-to-fascia distance (cm)			
Before device application (range)	13.5 (8–23)	17.5 (15–20)	*0.1361*
48 h at relook laparotomy after device application (range)	10 (6–17)	14 (7–15)	*0.0966*
Duration of OA prior to device application; days (range)	4.5 (1–14)	1.5 (0–5)	*0.2250*
Duration of OA until fascial closure after device application; days (range)	7.5 (3–24)	7 (6–12)	*0.8970*
Total duration of OA; days (range)	11.5 (5–28)	10.5 (6–13)	*0.8511*
Number of relook procedures until fascial closure; days (range)	4 (3–7)	3.5 (1–5)	*0.5983*

*h* hours, *OA* open abdomen

Interestingly, although the fascia-to-fascia distance of patients with non-septic OA (17.5 cm; range 15–20 cm) prior to VTD application was larger than that of patients with septic OA (13.5 cm; range 8–23 cm), both groups achieved similar primary fascial closure (PFC) period after device insertion (non-septic OA vs. septic OA: 7 days; range 6–12 days vs. 7.5 days; range 3–24 days; *p* = 0.8970) (Table 5). However, the mean duration of OA in patients with septic OA prior to VTD application was longer (4.5 days; range 1–14 days) than in patients with non-septic OA (1.5 days; range: 0–5 days). Data of this subgroup are summarized in Table 5.

## Discussion

In this study, a novel device that exerts a dynamic vertical traction force on the fascia was successfully implemented in a series of 20 patients. A major advantage of vertical traction that we observed was the ability to early prevent fascial retraction, especially in cases with increased intra-abdominal volume due to visceral edema. In our patient cohort, early PFC was achieved in all cases (100%) without mesh augmentation or component separation. No severe complications related to the novel device application were observed.

Early primary fascial closure is crucial to prevent the well-known complications of open abdomen (OA). Combinations of temporary abdominal closure systems with dynamic closure procedures have recently been reported to illustrate the best primary fascial closure (PFC) rates [2, 15, 18, 31, 32]. However, early application of dynamic closure procedures are restricted by various factors such as hemodynamic instability, uncontrolled source of infection, persistent abdominal peritonitis, or increased intra-abdominal volume due to visceral edema [13, 27]. Verdam et al. [13] and Reimer et al. [26] have previously reported an average duration of OA of 12 days and 18 days, respectively, prior to application of the abdominal re-approximation anchor system (ABRA). Reimer et al. reported a delayed primary fascial closure (fascia-to-fascia closure after 8 days of open abdomen, usually within the initial hospitalization [10]) rate of 61% for their mixed patient cohort (septic OA and non-septic OA), whereas Verdam et al. reported a delayed primary fascial closure rate of 88% in patients with advanced septic OA. In both reports, duration of OA treatment prior to application of the horizontal dynamic traction (ABRA) probably had an impact on primary closure rate. Verdam emphasized that timing to approximation could be an important factor for successful closure. In contrast to the above-mentioned studies, the mean duration of OA prior to the vertical traction device (VTD) implementation in our mixed patient cohort (12 septic OA and 8 non-septic OA) was 3 days (range 0–14 days), which was shorter compared to the two other studies. At this timing to approximation, we observed a successful closure rate of 100%. Two patients received the device directly at initial laparotomy due to an abdominal compartment syndrome (ACS) caused by a mechanical ileus. In all cases, early vertical dynamic traction exerted on the fascia by the device was possible without hemodynamic or respiratory function impairment. Additionally, despite bowel distension and visceral edema, dynamic traction was feasible without causing iatrogenic intra-abdominal hypertension. Only one patient received the device after 14 days of OA treatment due to infected, necrotizing pancreatitis. Our high closure rates highlight the fact that timing to dynamic approximation plays an important role for successful primary fascial closure.

In earlier studies, success rate of primary fascial closure has been reported to depend on the etiology of the open abdomen [2, 19, 20, 22]. In the systematic review and evidence-based recommendation for the use of negative pressure wound therapy (NPWT) by Brunhin et al. [2], patients with septic OA displayed lower fascial closure rates at the end of the therapy compared to patients with non-septic OA. Interestingly, when combined with a dynamic closure device, NPWT showed increased primary fascial closure rates. Verdam et al. [13] reported a delayed PFC rate of 88% for 14 patients with advanced septic OA (Björck grade 2B-4) after a mean OA duration of 25 days (range 7–48 days) using the dynamic traction of ABRA (abdominal re-approximation anchor system). Mintziras et al. [33] reported a delayed PFC rate of 47% for patients with secondary peritonitis under NPWT. In this study of Mintziras et al., the duration of OA until fascial closure was not analyzed. In a previous study by Tolonen et al. [17], a delayed PFC rate of 80% was reported over a median OA duration of 7 days in patients with secondary peritonitis treated with NPWT and a dynamic closure procedure (mesh-mediated fascial traction). Recently, Granger et al. [16] described a myofascial closure rate of 86.5% in patients with peritonitis treated mainly (97.7% of the cases) with ABThera™ dressing (NPWT with protective visceral layer). In this study, an average OA duration of 2.1 days was reported. These findings were in-line to our study in which twelve patients with septic OA (Björck grade 2B) were treated using the dynamic vertical traction device (VTD). Our primary PFC rate was 100% after a mean OA duration of 11.5 days (range 5–28 days). Although our OA duration was longer compared to previous studies [13, 17], we experienced higher closure rates as reported in the literature.

Similarly, for patients with non-septic open abdomen, total OA duration and primary fascial closure rates are heterogeneously reported. PFC rates vary from 65 to 100% and OA duration from 2.1 to 12.5 days [15, 16, 18, 21, 32]. In our cohort of eight patients with non-septic OA, PFC rate was 100% with a mean OA duration of 10.5 days (range 6–13 days). These results are better or in-line with recent reported studies cited above.

As previously mentioned, a temporary abdominal closure system in combination with a dynamic traction procedure has been reported to have best primary closure rates and enhance rapid fascial closure in both septic and non-septic open abdomen. In our case series, twelve patients were treated with a combination of the vertical traction device (VTD) and a negative pressure wound therapy (NPWT) (VTD-NPWT group), and eight patients received VTD in combination with an alternative temporary abdominal closure system (Bogotá bag and abdominal dressings) (VTD-TAC group). Prior to device application, the mean fascia-to-fascia (FTF) distance of patients in the VTD-NPWT group was significantly larger than the VTD-TAC group (17.5 cm vs. 13 cm, *p* = 0.0532). Interestingly, there was no significant difference between the duration of OA after the device was applied in both groups (VTD-NPWT vs. VTD-TAC: 7.5 days vs. 7 days; *p* = 0.8970). This result demonstrates the beneficial effect of NPWT in combination with a dynamic vertical traction device on treating large fascial defects.

One of our main objectives during clinical use of this device was to investigate whether the weight exerted by the device on the thorax and pelvis affected the patient’s hemodynamic and respiratory conditions, and whether pressure sores occurred. Additionally, we investigated whether intra-abdominal hypertension could occur when implementing this device. Therefore, these parameters were continuously monitored until the device was dismantled. One patient developed a grade one pressure sores according to Barczak et al. [30] at the position of the buttress on the anterior pelvic ring. The pressure sores were treated conservatively. Four patients developed a subcutaneous wound dehiscence 1 week after primary fascial closure (PFC), and two patients developed a fascial dehiscence leading to an incisional hernia 6 months after discharge. Our results revealed due to the high rate of PFC and the low rate of device-related complications that this device appears to be an effective tool in the treatment of OA.

This study has some major limitations due to its retrospective nature and the low number of patients. However, this is the first study to assess the clinical use of this novel device in a series of patients. This technique need to be validated in prospective controlled trial with a larger number of patients. Additionally, future studies should evaluate which patients might benefit from early vertical traction.

## Conclusion

Dynamic vertical traction prevented fascial retraction, enhanced abdominal wall extension, and thus facilitated early PFC in septic and non-septic open abdomen. In combination with negative pressure wound therapy (NPWT), vertical traction promoted rapid fascial closure of large abdominal defects. This could reduce the necessity of complex abdominal wall reconstruction as well as rate of mesh grafting and also mitigate morbidity and the socio-economic burden related to open abdomen treatment.

## Data Availability

The datasets used and/or analyzed during the current study are available from the corresponding author on reasonable request.
